# Public health threat of antimicrobial resistance and virulence genes in Escherichia coli from human-chicken transmission in Egypt

**DOI:** 10.1038/s41598-025-94177-w

**Published:** 2025-04-12

**Authors:** Zeinab S. Ahmed, Mahmoud E. Hashad, Yasser Atef, Heba Badr, Mahmoud Elhariri, Mona Kadry

**Affiliations:** 1 Department of Zoonoses, Veterinary Medicine, Cairo University, Giza, 12211 Egypt; 2 Department of Microbiology, Veterinary Medicine, Cairo University, Giza, 12211 Egypt; 3 Animal Health Research Institute, Giza, 12618 Egypt

**Keywords:** *E. coli*, Multi-drug resistance, Extended-spectrum β-lactamases, Virulence genes, Poultry, Microbiology, Antimicrobials, Bacteria, Infectious diseases

## Abstract

*Escherichia coli (E. coli)* infections cause significant losses in the poultry industry and pose zoonotic risks due to rising antimicrobial resistance (AMR) and virulence factors. This study investigates *E. coli* prevalence, AMR, and virulence genes (*papC*,* vgrG1*,* iss*) in Egyptian chickens and farm workers. A total of 35 dead chickens from 14 flocks and 17 farm workers urine samples were examined bacteriologically to investigate *E. coli* presence followed by biochemical identification. Antimicrobial susceptibility testing was performed on 14 antibiotics using the disk diffusion method on Mueller–Hinton agar, following ‘Clinical and Laboratory Standards Institute (CLSI) (2020) guidelines with Extended-spectrum β-lactamase (ESBL) activity evaluated via the Double Disc Synergy Test (DDST) with ceftazidime, cefotaxime, and their clavulanate combinations following CLSI protocols. virulence genes were detected through polymerase chain reaction (PCR) and phylogenetic analysis of the *vgrG1* gene evaluated genetic relatedness between the chicken and human isolates. The study analysed 52 samples, identifying *E. coli* in 18 chicken organs (51.4%) and 11 human urine samples (64.7%), with no significant difference. various antimicrobic sensitivity profiles were identified phenotypically among all isolates in which 29 isolates, 58.6% were ESBL-producing, and 96.5% exhibited multidrug resistance (MDR), with chicken isolates showing higher resistance overall. virulence genes were detected in similar proportions across the isolates highlighting significant public health risks due to resistant and virulent *E. coli*. This study emphasized the public health risks of multidrug-resistant *E. coli* with virulence genes, highlighting potential zoonotic transmission and antibiotic use and food safety.

**Supplementary Information:**

The online version contains supplementary material available at 10.1038/s41598-025-94177-w.

## Introduction

*Escherichia coli (E. coli)* infections are a major cause of significant financial losses in the global poultry industry, primarily due to decreased productivity, increased mortality, carcass condemnations, and the high costs of disease control^1–3^. As the causative agents of avian colibacillosis, one of the most prevalent diseases affecting poultry worldwide^4,5^. These virulent strains can induce a range of localized and systemic infections in chickens, including septicaemia, chronic respiratory disease, swollen-head syndrome, enteritis, cellulitis, salpingitis, omphalitis, synovitis, pericarditis, and peritonitis^6^.

In human, many *E. coli* strains are harmless and play a role in keeping gut health, but some are Pathogenic strains and classified into intestinal (IPEC) and extra-intestinal groups (ExPEC) which represents a broader group of pathotypes capable of causing infections outside the intestinal tract, such as urinary tract infections (UTIs) which ranging from asymptomatic bacteriuria to severe conditions like pyelonephritis and urinary-source bacteremia. Approximately half of all *E. coli* bloodstream infections originate from the urinary tract^7,8^.

Bacterial resistance is a worldwide threat to human, animal, and environmental health. If left unaddressed, it could lead to significant losses, decreased livestock production, and increased human mortality by 2050^9^. researchers have warned that cross-infections may become more widespread in developing countries due to factors such as the availability of illegal antibiotics, poor healthcare infrastructure, and environmental conditions conducive to the spread of resistant pathogens^10^.

Global reports, including from Egypt, highlight increasing resistance to antibiotics like β-lactams, fluoroquinolones, tetracyclines and aminoglycosides with Enterobacteriaceae developing resistance through the production of β-lactamases, particularly class A Extended-spectrum β-lactamase (ESBLs) and class C (*AmpC*) β-lactamases^11^. ESBLs render bacteria resistant to critical antibiotics, including third- and fourth-generation cephalosporins which have been considered critically important antimicrobials to human and veterinary medicine, and often result in co-resistance to other antibiotics, complicating treatment^12^.

The rise of antimicrobial resistance in commensal bacteria like *E. coli* poses a significant threat to public health as resistance patterns in animal *E. coli* often mirroring those in humans in addition to its ability to transfer antibiotic resistance genes to other pathogens^13,14^. The spread of ESBL-producing bacteria is affected by the overuse of antibiotics in humans, animals, and agriculture, with food-producing animals serving as key reservoirs. Resistant strains can enter the food chain through raw or undercooked meat, and bacteria from farms can contaminate the environment, spreading via air, water, soil, and pests like flies, which carry bacteria to human food^15^.

The emergence of antibiotic-resistant *E. coli* strains in poultry poses a growing challenge to controlling their spread with antibiotics. Key contributing factors include poor food hygiene, antibiotic misuse and overuse, and insufficient biosecurity and hygiene practices^16–18^. In regions like Egypt, where poultry is a major protein source, addressing the rise of AMR in food-producing animals, particularly poultry, is crucial to controlling this public health threat^19,20^.

The coexistence of antibiotic resistance and virulence factors in E. coli may enhance its pathogenicity and complicate treatment. Understanding the link between resistance patterns and virulence genes like *papC*, *vgrG1*, and *iss* is crucial, as their interaction could influence bacterial survival, infection severity, and therapeutic challenges^21^.

In addition to their possible resistance to antibiotics, strains of *E. coli* that are extra intestinally pathogenic have various virulence factors, or genes, which may combine to form a mix trait that makes the strain capable of causing illness. The *papC*,* vgrG1*, and *iss* genes in *E. coli* play important roles in the bacteria’s ability to cause and persist in the infection by enabling adherence to host cells (*papC*), promoting toxin secretion and cell invasion (*vgrG1*), and enhancing survival in the bloodstream by evading immune responses (*iss*), all of which play key roles in urinary tract and extraintestinal infections. all of which facilitate infection establishment and persistence, especially in severe cases^22–24^.

Despite many studies examining the prevalence of pathogenic *E. coli* in animals and human sources, limited research has focused on the co-occurrence of its antimicrobial resistance and virulence traits, particularly in Egypt. This study aims to investigate the prevalence of *E. coli* in chickens and farm workers in Egypt through analysing its antimicrobial resistance and virulence gene profiles assessing the potential zoonotic risks of severe, untreatable infections transmitted through the food chain.

## Materials and methods

The study received approval from the Institutional Animal Care and Use Committee at the Faculty of Veterinary Medicine, Cairo University. All methods were conducted in compliance with applicable rules and regulations.

### Samples collection and processing

Between December 2022 and November 2023, we examined 35 deceased chickens from 14 broiler flocks located in the Cairo, Giza, and Qalyubia Governorates, all of which were experiencing elevated mortality rates, respiratory symptoms, and diarrhea. Postmortem analyses revealed lesions associated with colisepticemia, including pericarditis, perihepatitis, and airsacculitis. We collected samples from the liver, lungs, heart, and spleen of these chickens for further investigation. The age of the chickens ranged from 25 to 30 days. Regarding antibiotic usage, the farms primarily administered antibiotics that were among those we selected for our research. Additionally, the farming system was open with medium stocking density. To assess potential zoonotic transmission, urine samples were also obtained from 17 farm workers who had direct contact with the affected birds. All samples were promptly transported to the laboratory under sterile, refrigerated conditions to maintain sample integrity.

### Isolation and identification of* E. coli*

Swabs from the collected organs and urine sediments were pre-enriched in brain-heart infusion broth and incubated aerobically at 37 °C for 24 h. After enrichment, a loopful of the broth was streaked onto Eosin Methylene Blue (EMB) agar and MacConkey agar (both from Oxoid, Hampshire, UK) and incubated aerobically at 37 °C for 24–48 h^11^. Colonies exhibiting distinct pink coloration on MacConkey agar and shiny, metallic green colonies on EMB agar were selected and subculture to obtain pure isolates. Morphological identification was conducted using Gram staining. Further biochemical characterization including the indole test, tests for urea hydrolysis, citrate utilization, and oxidase activity, as well as responses on triple sugar iron agar slants were used according to Nolan^2^ and Tille^25^.

### Antimicrobial susceptibility testing

The disk diffusion assay on Mueller–Hinton agar (Oxoid, Hampshire, UK) was performed to confirm the antibiotic sensitivity profile of the *E. coli* isolates following ‘Clinical and Laboratory Standards Institute guidelines (CLSI, 2020). A panel of 14 antibiotic discs were selected based on their frequent use in both human and veterinary medicine. These discs represent different antimicrobial classes: β-lactams (Penicillins: Ampicillin (AMP;10 µg) Cephalosporins: cefotaxime (CTX; 30 µg), ceftazidime (CAZ; 30 µg) and Ceftriaxone (CTR; 30 µg), beta-lactam combination as amoxicillin/ clavulanic acid (AMC; 20/10 µg), Aminoglycosides (gentamycin (CN; 10 µg), Fluorquinolones (Ciprofloxacin (CIP; 5 µg), Quinolones (nalidixic acid (NA; 30 µg), Tetracycline ( doxycycline (DO; 30 µg), tetracycline (TE; 30 µg), Sulfonamides (Trimethoprim/Sulfamethoxazole (SXT; 1.25 µg/23.75 µg), and Phenicols (Chloramphenicol (C; 30 µg), Nitrofuran (nitrofurantoin (NIT; 30 µg). Polymyxins: colistin (COL; 10 µg)^26^.

The isolates were classified based on the inhibition zone diameters as resistant, intermediate or susceptible according to the CLSI (2020). Bacteria that are resistant to at least one antimicrobial agent in three or more antimicrobial groups are known as multi-drug-resistant (MDR) bacteria. The reference strain *Escherichia coli* ATCC 25922was run in parallel to test the assay reliability.

### Synergy-based ESBL activity detection

To evaluate the potential extended-spectrum β-lactamase (ESBL) activity of the *E. coli* isolates, the Double Disc Synergy Test (DDST) was performed using antibiotic discs, including Ceftazidime (CAZ, 30 µg), Ceftazidime-clavulanate (CAZ-CL, 30 µg/10 µg), Cefotaxime (CTX, 30 µg), and Cefotaxime-clavulanate (CTX-CL, 30 µg/10 µg). The test was carried out following Clinical and Laboratory Standards Institute (CLSI) guidelines. Confirmation of ESBL production was based on the protocols described by^27,28^.

### Molecular detection of virulence genes

PCR was working to detect the presence of the *pap*C, *vgr*G1, and *iss* genes in 29 *E. coli* isolates. Bacterial DNA was extracted from the isolates using a traditional boiling method, as described by Ibrahim et al.^6^. The target genes, oligonucleotide primer sequences, the product size and thermocycling conditions are listed in Table 1. Each PCR reaction had a final volume of 25 µl, composed of 3 µl of extracted DNA, 12.5 µl of master mix (EmeraldAmp GT PCR, Takara, Japan), 0.5 µl of 10 µM of each primer, and 8 µl of nuclease-free water (Qiagen, Germany). PCR amplification was conducted using a heat cycler (Techne^®^ Prime, UK). All PCR products were electrophoresed on a 1.5% agarose gel and visualized using a UV transilluminator. A 100 bp DNA ladder (with a size range of 100–1000 bp; Jenna Bioscience GmbH, Jenna, Germany) was run alongside the samples to estimate the size of the PCR amplicons.

**Table 1:** Primer sequences, amplicon sizes, and PCR conditions of the* Escherichia coli* virulence genes.

Reference	Target gene	Primer sequence (5ʹ–3ʹ)	Amplicon size (bp)	PCR conditions
^35^	*Iss*	F: ATGTTATTTTCTGCCGCTCTG R: CTATTGTGAGCAATATACCC	266	initial denaturation of 5 min at 94 ◦C followed by 35 cycles, denaturation at 94 ◦C for 30 s, annealing at 54 ◦C for 30 s, extension at 72 ◦C for 30 s, and final extension at 72 ◦C for 10 min.
^48^	*pap*C	F: GACGGCTGTACTGCAGGGTGTGGCG R: ATATCCTTTCTGCAGGGATGCAATA	350	initial denaturation of 5 min at 94 ◦C followed by 30 cycles, denaturation at 94 ◦C for 40 s, annealing at 65 ◦C for 40 s, extension at 72 ◦C for 1 min, and final extension at 72 ◦C for 10 min
^49^	*vgr*G1	F: GTATCTTCCAGAATGAGGAC R: CATGTTCATCACAGAAGATT	831	initial denaturation of 5 min at 94 ◦C followed by 25 cycles, denaturation at 94 ◦C for 30 s, annealing at 54 ◦C for 30 s, extension at 72 ◦C for 1 min, and final extension at 72 ◦C for 10 min

### Statistical analysis

The chi-square test (χ2) was conducted to determine the correlation among the *E. coli* isolates occurrences in the various samples. Statistics were considered significant when *p* < 0.05.

### Sequencing and phylogenetic analysis

The *vgrG1* gene PCR products from randomly selected *E. coli* isolates (one from a urine sample and another from chicken organ samples) were purified using the Gene JET PCR Purification Kit (Thermo), following the manufacturer’s instructions. Sequencing was conducted using a DNA sequencer and the Big Dye Terminator V3.1 Cycle Sequencing Kit (Applied Biosystems). The resulting sequences were analysed using BLAST to compare them with those available in the GenBank database (NCBI). Phylogenetic tree analysis was performed to evaluate the genetic relatedness of *vgrG1* gene sequences obtained from human and chicken sources. Sequence alignment and identity matrices were generated using the Clausthal W multiple alignment tool in the BioEdit program. Phylogenetic trees were constructed using the neighbour-joining method with the MEGA6 software (version 6.06).

## Results

### Occurrence of* E. coli* in the examined samples

A total of 52 samples, comprising 35 chicken organ samples and 17 human urine samples as illustrated in Table 2 were analysed for *E. coli* isolation. Among these, 18 isolates (51.4%) were recovered from chicken organs and 11 isolates (64.7%) from human urine samples, resulting in an overall isolation rate of 55.8%. Statistical analysis indicated no significant difference between the isolation rates from chicken and human samples (*p* > 0.05). (Table 3).

**Table 2:** Occurrence of* Escherichia coli* in chicken organs and human urine samples.

Sample type	Number of samples	Number of isolates (%)	p-value
Chicken organs	35	18 (51.4)	> 0.05
Human urine	17	11(64.7)	
Total	52	29(55.8)	

X^2^ The chi-square: the result is not significant at p > 0.05.

### Phenotypic detection of ESBL-producing and non-ESBL-producing* E. coli*

The Double Disc Synergy test (DDST) shown that out of 29 *E. coli* isolates, 17 (58.6%) were ESBL-producing, while 12 (41.3%) were non-ESBL-producing as detailed in Table 4. Among the chicken organ isolates, 11/18 (61.1%) were ESBL producers, compared to 6/11 (54.5%) in human urine samples. Non-ESBL-producing strains accounted for 7/18 (38.8%) of chicken isolates and 5/11 (45.4%) of human isolates. No significant difference was observed in the distribution of ESBL production between the sample types (*p* > 0.05). (Table 5).

### Antimicrobial susceptibility profile in the* E. coli* isolates

In chicken isolates (*n* = 18) exhibited 100% resistance to penicillins (AMP and AMC) and phenicols (C), with high resistance also observed against cephalosporins (CTX: 94.4%, CAZ: 100%), tetracyclines (DO: 88.8%, TE: 94.4%), and sulfonamides (SXT: 94.4%). Fluoroquinolone (CIP) and quinolone (NA) resistance was 72.2% and 88.8%, respectively. Aminoglycosides (CN) and polymyxins (COL) showed moderate resistance at 33.3% and 38.8%, respectively. In human isolates (*n* = 11), resistance was also notable for penicillins (AMP: 81.8%, AMC: 100%) and cephalosporins (CTX: 63.6%, CAZ: 72.7%), but much lower for tetracyclines (TE: 18.1%, DO: 9.1%), sulfonamides (SXT: 18.1%), and polymyxins (COL: 9.1%). Some classes, such as quinolones (NA) and phenicols (C), showed no resistance among human isolates. Overall, chicken isolates exhibited higher resistance across most antibiotic classes compared to human isolates. in general, 28 of the 29 *E. coli* isolates (96.5%) revealed multidrug resistance (MDR) and extensively drug-resistant (XDR) phenotype while no isolates showed pan-drug-resistant (PDR) characteristics (Tables 3 and 6 and S1).

**Table 3:** Antimicrobial resistance among* Escherichia coli* isolates from chicken and human samples.

Antibiotic class	Antibiotic (concentration)	Resistant strains chickens (*N* = 18)	Total (*N* = 29) humans (*N* = 11)
Penicillins	AMP (10 µg)	18 (100%)	9(81.8%)
	AMC (20/10 µg)	18(100%)	11(100%)
Cephalosporins	CTX (30 µg)	17(94.4%)	7(63.6%)
	CAZ (30 µg)	18(100%)	8(72.7%)
	CTR (30 µg)	15(83.3%)	0(0%)
Aminoglycosides	CN (10 µg)	6(33.3%)	1(9.1%)
Fluoroquinolones	CIP (5 µg)	13(72.2%)	2(%)
Quinolones	NA (30 µg)	16(88.8%)	0(%)
Tetracyclines	DO (30 µg)	16(88.8%)	1(9.1%)
	TE (30 µg)	17(94.4%)	2(18.1%)
Sulfonamides	SXT (25 µg)	17(94.4%)	2(18.1%)
Phenicols	C (30 µg)	18(100%)	0(0%)
Polymyxins	COL (10 µg)	7(38.8%)	1(9.1%)
Nitrofuran	NIT (30 µg)	10(55.5%)	0(0%)

**Table 4:** Phenotypic ESBL-producing and non-ESBL-producing* Escherichia coli* in chicken organs and human urine samples.

Sample type	ESBL-producing* E. coli*	Non-ESBL-producing* E. coli*	*p*-value
Chicken organs	11/18 (61.1%)	7/18 (38.8%)	0.182
Human urine	6/11(54.5%)	5/11 (45.4%)	0.189
Total	17/29 (58.6%)	12/29 (41.3%)	

The result is not significant at *p* > 0.05.

**Table 5:** Occurrence of* papC*,* iSS*, and* vgrG1* virulence genes among Escherichia coli isolates.

Virulence genes	Chicken isolates (*n* = 18)	Human isolates (*n* = 11)	Total (*n* = 29)	Chi-square (Xآ²)
*papC*	9 (50%)	6 (54.5%)	15 (51.7%)	0.0565
*iSS*	10 (55.5%)	7 (63.6%)	17 (58.6%)	0.1838
*vgrG1*	7 (38.8%)	4 (36.3%)	11 (37.9%)	0.0185

The result is not significant at *p* > 0.05.

### Occurrence of* papC*,* iSS*, and* vgrG1* virulence genes among* E. coli* isolates

In Table 5 in this study, the PCR of *papC*,* iSS*, and *vgrG1* virulence genes in 29 *E. coli* isolates revealed that *papC* gene was present in 9(50%) of chicken isolates and 6(54.5%) of human isolates; *iSS* gene was found in 10(55.5%) of chicken isolates and 7(63.6%) of human isolates; and *vgrG1* gene occurred in 7(38.8%) of chicken isolates and 4(36.3%) of human isolates. The overall occurrence of the three genes across both groups was 15(51.7%), 17(58.6%), and 11(37.9%), respectively. Statistical analysis using the chi-square test showed no significant differences between the chicken and human isolates for any of the genes with all p-values exceeding 0.05. These results indicate that the distribution of these virulence genes is comparable across the chicken and human *E. coli* isolates, suggesting no strong association with the origin of the isolates.

### Antibiotic resistance profiles, ESBL production, and virulence genes correspondence in* E. coli* isolates

The study discussed the antimicrobial resistance profiles and corresponding virulence gene patterns of 29 *E. coli* isolates, derived from both chickens and human origins as showed in Table 6. Each isolate’s antibiotic resistance profile is listed alongside the presence of ESBL production and the associated virulence genes. In total, 29 isolates exhibit varying combinations of resistance to multiple antibiotics, with specific virulence genes like *papC*,* iSS*, and *vgrG1* associated with these profiles. Notably, a few isolates test positive for ESBL production, with certain virulotypes corresponding to more complex resistance patterns involving multiple antibiotics. The *iss* gene was present in 7 isolates, accounting for 24.14% of the total. The *papC* and *vgrG1* genes were found in 9 (31%) and 2 (6.9%) isolates, respectively. Co-occurrence of the *iss* and *papC* genes was observed in 2 isolates (6.9%), while both *iss* and *vgrG1* genes were present together in 5 isolates (17.2%). Additionally, one isolate (3.4%) carried both *papC* and *vgrG1*, and three isolates (10.3%) harboured all three genes. The results reflect a diversity of resistance and virulence profiles in the same isolates, indicating that these bacteria are both resistant to antibiotics and more capable of causing disease, posing a significant public health risk.

**Table 6:** Antibiotic resistance profiles, ESBL production, and virulence gene correspondence in* Escherichia coli* isolates.

Antibiotic resistance profile	No. of isolates	Frequency (%)	ESBL- producer	Corresponding virulence genes
AMC, CIP	1	3.4		*pap*C
AMC, AMP, CAZ	2	6.9		*pap*C
AMC, CTX, CAZ	1	3.4		*iSS*
AMC, AMP, CN	1	3.4		*vgr*G1, *pap*C
AMC, AMP, CTX, CAZ	2	6.9		*iSS*,* vgrG*1, *papC*
AMC, AMP, COL, TE, CTX	1	3.4		*pap*C
AMC, AMP, SXT, CTX, CAZ	1	3.4		*iSS*
AMC, AMP, TE, SXT, CTX, CAZ	1	3.4	*+*	*vgr*G1, *papC*,* iSS*
AMC, AMP, C, CTX, CTR, CAZ	2	6.9		*iSS*,* vgr*G1
AMC, AMP, DO, C, TE, NIT, COL, SXT, CAZ	1	3.4		*pap*C
AMC, AMP, DO, C, TE, NA, SXT, CTX, CTR, CAZ	1	3.4	*+*	*iSS*
AMC, AMP, DO, C, TE, NA, SXT, CTX, CTR, CAZ	1	3.4	*+*	*papC*
AMC, AMP, DO, C, TE, NA, SXT, CIP, CTX, CTR, CAZ	3	10.3	*+*	*iSS*
AMC, AMP, DO, C, TE, NIT, NA, SXT, CIP, CTX, CTR, CAZ	2	6.9	*+*	*vgrG1*
AMC, AMP, DO, C, TE, CN, NA, SXT, CIP, CTX, CTR, CAZ	2	6.9	*+*	*papC*,* iSS*
AMC, AMP, DO, C, TE, NIT, NA, COL, SXT, CIP, CTX, CTR, CAZ	3	10.3	*+*	*papC*
AMC, AMP, DO, C, TE, CN, NIT, NA, SXT, CIP, CTX, CTR, CAZ	1	3.4	*+*	*iSS*
AMC, AMP, DO, C, TE, CN, NIT, NA, COL, SXT, CIP, CTX, CTR, CAZ	3	10.3	*+*	*iSS*,* vgrG1*

## Discussion

*E. coli*, a major foodborne pathogen, poses a critical challenge to food safety and public health globally. In this study, *E. coli* was detected in chicken organ and human urine samples, with an overall occurrence rate of 55.8%. These findings align with previous studies, such as those by Ibrahim et al.^6^. and Khalaf et al.^29^ which reported *E. coli* isolation rates from Egyptian chickens. The comparable prevalence of *E. coli* in chicken and human samples observed in this study suggests potential risk factors, including similar environmental exposures or overlapping transmission pathways^6^.

Antimicrobial resistance has been recognized as an emerging worldwide problem and antibiotic-resistant *E. coli* have documented in various studies, the study highlights the significant epidemiological concern of antimicrobial resistance (AMR) in *E. coli* isolates from both chickens and humans. In chicken isolates, 100% resistance was observed against AMP, AMC, C, and CAZ, and over 94% resistance to TE, SXT, and CTX. These findings are consistent with studies by Aberkane et al.^28^., Hasona et al.^31^ and Awad et al.^32,33^. who also reported significant resistance to AMP, and Hamed et al.^32^, who found similar resistance to CAZ. Moderate resistance rates were noted for DO (88.8%), NA (88.8%), CTR (83.3%), and CIP (72.2%). Lower resistance was seen in COL and CN. Similarly, other studies, such as Awad et al.^32^ and Ammar et al.^35^ found similar resistance patterns for AMC, while Abdel-Rahman et al.^36^ and Halfaoui et al.^37^ documented moderate resistance levels, particularly for antibiotics like AMC and CAZ.

In human isolates, resistance was notably lower, with high resistance only for AMC, AMP, CAZ, and CTX. Fluoroquinolones and quinolones showed variable resistance, with high resistance in chicken isolates to NA and CIP but much lower rates in human isolates. These findings highlight significant differences in antibiotic exposure and usage between poultry and humans, with chickens being subjected to higher antibiotic pressures. The study emphasizes the urgent need for improved antibiotic uses in both veterinary and human medicine. Also, colistin resistance was observed at 38.8% in chicken isolates and 9.1% in human isolates, COL resistance is relatively uncommon and unusual but raising alarms about the misuse of this critical last-resort antibiotic in poultry which poses a major public health risk^38^. Colistin resistance should be confirmed using the broth microdilution method, which represents a limitation of our study.

Globally, *E. coli* MDR rates have changed and increased over the past few decades^39^. This study illustrated 96.5% of isolates classified as MDR, and XDR phenotypes, while none demonstrated PDR characteristics (Table S1). the results emphasize the risks of excessive antibiotic use, which promotes the emergence and spread of resistant strains through direct contact or the food chain. The One Health approach is vital for mitigating the risks posed by MDR *E. coli* and protecting both public health and animal welfare.

The results from the Double Disc Synergy Test (DDST) reveal significant insights into the prevalence of Extended-Spectrum Beta-Lactamase (ESBL)-producing *E. coli* isolates in both chicken and human samples. Of the 29 *E. coli* isolates tested, 58.6% were ESBL-producing which is a concerning finding, as ESBL-producing bacteria are known to resist the action of commonly used beta-lactam antibiotics, including penicillins and cephalosporins^40^. Also, the production of beta-lactamase enzymes, including ESBLs is a key driver of the spread of multidrug-resistant (MDR) high-risk clonal lineages in which CTX-M-like enzymes are the most common, especially in *E. coli* isolated from animals and humans^41^.

The proportion of ESBL producers was slightly higher in chicken isolates compared to human urine samples. the higher proportion of ESBL-producing *E. coli* in poultry could be a sign of more selective pressure on these bacteria, promoting the emergence and persistence of resistant strains^42^. and study by Husna, A., et al.^43^ concluded that antibiotic resistance, particularly the production of ESBLs, is relatively more prevalent in the poultry population than in humans which could be due to the higher levels of antibiotic use in the poultry industry, including the routine use of antibiotics for growth promotion and disease prevention which then spread within the poultry population and potentially to humans through the food chain or direct contact.

Uropathogenic *E. coli* (UPEC), a pathotype of extraintestinal pathogenic *E. coli* (ExPEC), is a leading cause of urinary tract infections (UTIs) in humans and animals globally, with its virulence genes playing a central role in initiating both intestinal and extraintestinal infections, driving significant worldwide research interest^40^. this study examined the presence of three virulence genes (*papC*,* iSS*, and *vgrG1*) in *E. coli* isolates from chickens and humans. The *papC* gene was found in 50% of chicken isolates and 54.5% of human isolates, the *iSS* gene in 55.5% and 63.6%, and the *vgrG1* gene in 38.8% and 36.3%, respectively. This finding aligns with results by Mohammed et al.,^11^ and Hamelin et al.,^44^ who stated that greatest *E. coli* isolates were ExPEC pathotypes.

The findings showed that *E. coli* strains from chickens and humans share similar distributions of the *papC*,* iSS*, and *vgrG1* virulence genes, which suggests potential cross-species transmission, a notable concern in zoonotic diseases. This overlap indicates that certain *E. coli* strains may act as opportunistic pathogens in both humans and animals, capable of causing infections such as UTIs and gastrointestinal illnesses, regardless of the host species^45^. and such observation concluded that UTIs in humans may increase through exposure to contaminated poultry products, highlighting the need for enhanced monitoring of virulence genes, improved hygiene practices, and stricter food safety measures to prevent the transmission of pathogenic strains from animal reservoirs.

The results reveal a complex relationship between antimicrobial resistance profiles, ESBL production, and virulence genes in *E. coli* isolates from chickens and humans. Among the 29 isolates, ESBL producers accounted for 41.4% of the isolates, varying resistance patterns to multiple antibiotics (involving 10 or more antibiotics) were observed and often linked to specific virulence genes such as *papC*,* iSS*, and *vgrG1*. Co-occurrence of virulence genes was noted in several isolates, with 10.3% harbouring all three genes, reflecting a heightened pathogenic potential. The positive correlation between ESBL production and complex resistance profiles in some isolates underscores their dual capability to resist treatment and cause disease^44^. These findings highlight the significant public health risks posed by these multidrug-resistant and virulent *E. coli* strains, emphasizing the need for alert monitoring and control measures to mitigate their spread.

**Fig. 1:**
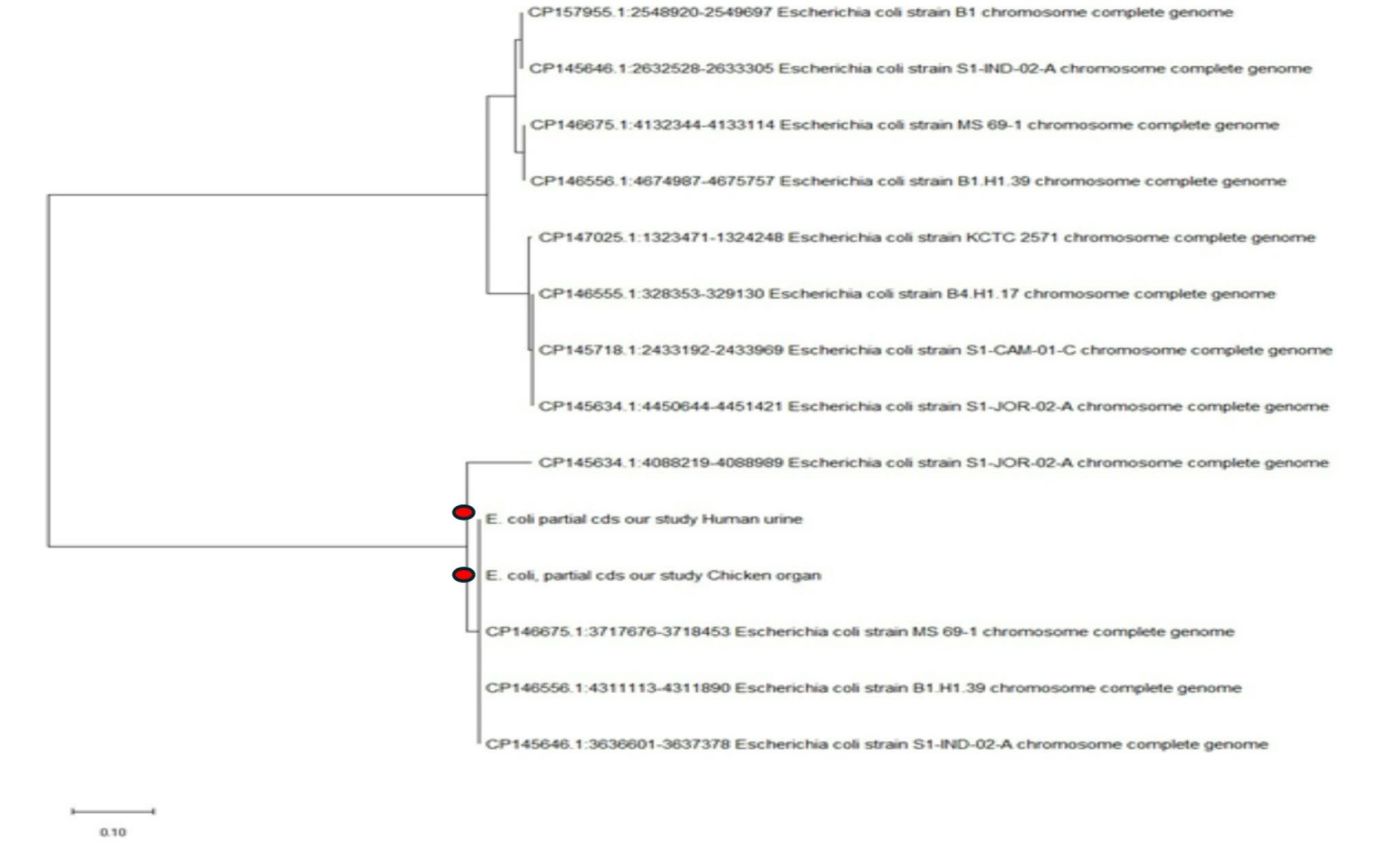
A neighbour-joining phylogenetic tree was constructed with MEGA X software, version 10.2.0. using nucleotide sequences of two *vgrG1* genes from* Escherichia coli* isolates. The isolates included in this study were obtained from chicken organs and human urine samples. The studied sequences were remark by bullets.

The neighbour-joining phylogenetic tree illustrates the genetic relationships between *E. coli* isolates based on their *vgrG1* gene sequences. The study’s isolates, derived from chicken organs (PQ399679) and human urine (PQ399678), are shown alongside other isolates retrieved from GenBank (Fig. 1) forming a distinct cluster suggesting a close genetic relationship between these isolates, which could indicate potential zoonotic transmission or shared evolutionary origins^46^.

A remarkable observation is that the proximity of isolates from human and chicken sources in the same clade suggests that *E. coli* strains harbouring the *vgrG1* gene, linked to bacterial virulence and infection, may circulate between humans and chickens. This aligns with the idea that poultry may serve as a reservoir for pathogenic *E. coli* strains that could infect humans^47^.

## Conclusion

This study highlights the significant public health risks posed by *E. coli*, a major foodborne pathogen, with high prevalence rates in both chicken and human samples, suggesting the potential transmission between humans and poultry. The findings reveal alarming multidrug-resistant (MDR) *E. coli* with producing extended-spectrum beta-lactamases (ESBLs), reflecting the overuse of antibiotics in poultry farming and underlining the growing threat of resistant strains. The detection of virulence genes (*papC*,* iSS*, and *vgrG1*) points to potential cross-species transmission, raising concerns about zoonotic infections like UTIs. Further studies with larger sample sizes are needed to confirm significant differences between groups. The study emphasizes the critical importance of implementing stringent biosecurity measures in poultry farming to mitigate the risk of pathogen transmission between poultry and humans. Key practices include maintaining strict personal hygiene, such as thorough handwashing before and after handling birds; utilizing protective clothing like disposable boot covers; restricting farm access to essential personnel; and establishing regular cleaning and disinfection schedules for equipment and vehicles. Identifying critical control points, such as human-animal interactions, sanitation practices, and antibiotic usage monitoring, is vital for effective biosecurity. Collectively, these protocols significantly reduce the risk of pathogen transmission between poultry and humans.

## Electronic supplementary material

Below is the link to the electronic supplementary material.

Supplementary Material 1

## Data Availability

All the data generated or analyzed in this study are included in this published article.
