# Supplementary material for: Public health threat of antimicrobial resistance and virulence genes in Escherichia coli from human-chicken transmission in Egypt
Source: Sci Rep. 2025 Apr 12;15:12627. doi: 10.1038/s41598-025-94177-w (PMC11993692; doi:10.1038/s41598-025-94177-w)
Supplement: Supplementary file 1 — Supplementary Material 1 [file 41598_2025_94177_MOESM1_ESM.docx]

| E. coli isolates | β-lactams | | | | β-lactams combination | Tetracycline | | Phenicols | Aminoglycosides | Nitrofuran | Quinolones | Polymyxins | Sulfonamides | Fluoroquinolones | MDR | XDR | PDR | ESBL | virulent genes |
| --- | --- | --- | --- | --- | --- | --- | --- | --- | --- | --- | --- | --- | --- | --- | --- | --- | --- | --- | --- |
|  | penicillin | Cephalosporins | | |  |  |  |  |  |  |  |  |  |  |  |  |  |  |  |
|  | AMP | CTX | CTR | CAZ | AMC | DO | TE | C | CN | NIT | NA | COL | SXT | CIP |  |  |  |  |  |
| 1 | R | R | R | R | R | R | R | R | R | R | R | R | R | R | + | + | - | + | Iss,vgr |
| 2 | R | R | R | R | R | R | R | R | R | R | R | R | R | R | + | + | - | + | Iss,vgr |
| 3 | R | R | R | R | R | R | R | R | R | R | R | R | R | R | + | + | - | + | Iss,vgr |
| 4 | R | R | R | R | R | R | R | R | R | R | R | S | R | R | + | + | - | + | iss |
| 5 | R | R | R | R | R | R | R | R | S | R | R | R | R | R | + | + | - | + | papc |
| 6 | R | R | R | R | R | R | R | R | S | R | R | R | R | R | + | + | - | + | Papc |
| 7 | R | R | R | R | R | R | R | R | S | R | R | R | R | R | + | + | - | + | Papc |
| 8 | R | R | R | R | R | R | R | R | R | S | S | R | R | R | + | + | - | + | Papc,iss |
| 9 | R | R | R | R | R | R | R | R | R | S | S | R | R | R | + | + | - | + | Papc,iss |
| 10 | R | R | R | R | R | R | R | R | S | R | S | R | R | R | + | + | - | + | Vgr |
| 11 | R | R | R | R | R | R | R | R | S | R | S | R | R | R | + | + | - | + | Vgr |
| 12 | R | R | R | R | R | R | R | R | S | S | R | S | R | R | + | + | - | + | Iss |
| 13 | R | R | R | R | R | R | R | R | S | S | R | S | R | R | + | + | - | + | Iss |
| 14 | R | R | R | R | R | R | R | R | S | S | R | S | R | R | + | + | - | + | Iss |
| 15 | R | R | R | R | R | R | R | R | S | S | R | S | R | S | + | + | - | + | Papc |
| 16 | R | R | R | R | R | R | R | R | S | S | R | S | R | S | + | + | - | + | Iss |
| 17 | R | S | S | R | R | R | R | R | S | R | S | R | R | S | + | + | - | - | Papc |
| 18 | R | R | R | R | R | S | S | R | S | S | S | S | S | S | + | + | - | - | Iss,vgr |
| 19 | R | R | R | R | R | S | S | R | S | S | S | S | S | S | + | + | - | - | Iss,vgr |
| 20 | R | R | S | R | R | S | R | S | S | S | S | S | R | S | + | + | - | + | Iss.vgr,papc |
| 21 | R | R | S | R | R | S | S | S | S | S | S | S | R | S | + | + | - | - | Iss |
| 22 | R | R | S | S | R | S | R | S | S | S | S | R | S | S | + | + | - | - | Papc |
| 23 | R | R | S | R | R | S | S | S | S | S | S | S | S | S | + | + | - | - | Iss,vgr,papc |
| 24 | R | R | S | R | R | S | S | S | S | S | S | S | S | S | + | + | - | - | Iss,vgr,papc |
| 25 | R | S | S | S | R | S | S | S | R | S | S | S | S | S | + | + | - | - | Vgr,papc |
| 26 | S | R | S | R | R | S | S | S | S | S | S | S | S | S | + | + | - | - | Iss |
| 27 | R | S | S | R | R | S | S | S | S | S | S | S | S | S | + | + | - | - | Papc |
| 28 | R | S | S | R | R | S | S | S | S | S | S | S | S | S | + | + | - | - | papc |
| 29 | S | S | S | S | R | S | S | S | S | S | S | S | S | R | + | + | - | - | papc |

**Phenotypic resistance profile Among *Escherichia coli* isolates from Chicken and Human samples.**
